# Influence of a Multifaceted Nursing Practice Bundle to Support Hospitalised Adults With Cognitive Impairment: A Longitudinal Qualitative Study

**DOI:** 10.1111/jocn.70404

**Published:** 2026-06-22

**Authors:** Erin Rachel Davis, Christina Aggar, Michelle Bissett

**Affiliations:** ^1^ Southern Cross University Bilinga Queensland Australia; ^2^ Northern NSW Local Health District Lismore New South Wales Australia

## Abstract

**Aim:**

To explore nurses' perceptions of the influence of a multifaceted nursing practice bundle, and the factors affecting practice change over time.

**Design:**

A longitudinal qualitative study with recurrent cross‐sectional analysis over two timepoints.

**Methods:**

Thematic analysis of focus groups and interviews with nurses.

**Results:**

17 nurses participated, and four themes emerged. Two described how the bundle influenced nursing practice: *supporting positive nonpharmacological practices* and *strengthening communication, collaboration, and relationships.* Two addressed the factors affecting practice change: *leadership driving practice change* and *purpose‐driven nursing*.

**Conclusion:**

Nurses reported a positive influence of the bundle on practice. Leadership and the bundle's strong alignment with nurses' professional and personal values appeared to drive perceived practice change over time.

**Implications for the Profession:**

This study highlights the potential of a multifaceted nursing practice bundle to enhance care for adults with cognitive impairment in hospital. The multifaceted approach may facilitate adoption across health services, with ongoing practice change driven by supportive leadership and alignment with nurses' values.

**Impact:**

Nonpharmacological interventions are internationally recommended to support adults with cognitive impairment, but are underutilised by nurses in hospital. Nurses described enhanced practice following implementation of the bundle, with leadership and alignment to nurses' values perceived as key drivers of practice change. This study provides recommendations for organisation‐led programs to support nursing practices and improve the quality of care for hospitalised adults with cognitive impairment.

**Reporting Method:**

Reported in accordance with COREQ.

**Patient or Public Contribution:**

No direct patient or public contribution.

## Key Points

- What does this paper contribute to the wider global clinical community?
  ○ There is limited evidence exploring programs aimed at enhancing nursing practice to support adults with cognitive impairment in hospitals. This study is the first longitudinal qualitative exploration completed from the perspective of nurses.
- Adults with cognitive impairment experience poorer hospital outcomes, often linked to behaviours that challenge arising from unmet needs.
- Nonpharmacological interventions may address unmet needs, with nurses well placed to deliver them.
- A multifaceted nursing practice bundle may improve nursing practice, including nonpharmacological interventions.
- Findings highlight the importance of aligning organisational structures with nursing values.

## 1 Introduction

The presence of cognitive impairment in adults, most commonly dementia, delirium, or both, is a predictor of increased acute care needs and risk of hospitalisation (Australian Commission on Safety and Quality in Health Care [ACSQHC] 2019). During hospitalisation, this patient cohort is at substantially higher risk of adverse events, including falls, pressure injuries, and infection, leading to longer length of stay, functional decline, and increased mortality (ACSQHC 2023; Fogg et al. 2018). Inadequate nursing responses to behaviours that challenge, such as aggression, agitation, and refusal of care, contribute to preventable complications (Anantapong et al. 2025; Ye et al. 2024). Person‐centred nonpharmacological interventions implemented at the early signs of distress are central to preventing escalation and improving patient experiences for adults with cognitive impairment (Carrarini et al. 2021; James et al. 2023). To support the effective use of these interventions in practice, organisationally initiated programs that strengthen nurses' knowledge and skills are needed (Casarez and Smith 2024).

## 2 Background

For adults with cognitive impairment, behaviours that challenge may occur and escalate in response to physical and psychological stressors, which supersede a biologically lowered stress threshold related to neurological changes (Hall and Buckwalter 1987; Pickering et al. 2022). In hospital settings, these stressors include changes in caregivers, routines, and environments, as well as illness exacerbation (Pickering et al. 2022) and unmet biological and social needs (Cohen‐Mansfield et al. 2015). The Cohen‐Mansfield unmet needs model conceptualises these behaviours as substitutes for traditional communication in adults with cognitive impairment seeking assistance (Cohen‐Mansfield 2000). Nurses' understanding of behavioural aetiology directly impacts nursing practice (Casarez and Smith 2024; Çevik et al. 2022), with greater knowledge associated with increased adherence to international recommendations for person‐centred nonpharmacological interventions (ACSQHC 2019; National Institute for Health and Care Excellence [NICE], 2010/ 2023; NICE 2018; World Health Organisation 2017).

International guidelines recommend person‐centred nonpharmacological interventions as first‐line strategies for preventing and managing behaviours that challenge, given their low risk and effectiveness in reducing stressors and addressing unmet needs (ACSQHC 2019; NICE 2018). Despite this, high‐risk restrictive practices remain common in hospitals, often suppressing behaviour without addressing underlying causes (Ma et al. 2020). Education that strengthens nurses' understanding of patient needs and demonstrates the effectiveness of nonpharmacological approaches has been shown to positively shift practice, particularly when supported by implementation frameworks and nonpharmacological resources (Abbott et al. 2022; Carrarini et al. 2021; Casarez and Smith 2024; Ma et al. 2020; Jesto et al. 2024). Accordingly, a multifaceted nursing practice bundle that incorporates an implementation framework, education, and nonpharmacological resources may enhance nursing care and improve hospitals' outcomes for adults with cognitive impairment. However, the evidence surrounding this is limited.

Practice‐change initiatives often fail to demonstrate impact over time, due to limited resources, inadequate leadership, and competing time demands in hospital, with nursing engagement likely key (Alexander et al. 2022; Cowie et al. 2020; Ye et al. 2024). Acknowledging the complexities of acute and subacute care settings, a longitudinal qualitative exploration of nurses' perceptions of the influence of a multifaceted nursing practice bundle and the factors affecting practice change over time is needed to inform future implementation strategies.

## 3 The Study

### 3.1 Intervention—A Multifaceted Nursing Practice Bundle

A multifaceted nursing practice bundle, co‐designed by researchers, nurses, patients, and family carers, was developed to better support hospitalised adults with cognitive impairment. Three elements were included: (i) a health district‐initiated implementation framework (File S1), (ii) a nurse education package, and (iii) nonpharmacological resources (including fidget mats and boards). The framework guides nursing practice by categorising commonly observed behaviours into four stages of escalation (mildly confused, mild agitation, moderate agitation, and severe agitation) and by providing person‐centred nonpharmacological nursing strategies to support patients (Northern New South Wales Local Health District 2022). A single‐sided poster of the framework was distributed to the wards. Nurse education consisted of a 30‐min face‐to‐face session delivered by specialist nurses, accompanied by a PowerPoint presentation (with verbal script) to support standardised delivery. The education outlined the relationship between unmet needs and behaviours that challenge in adults with cognitive impairment, and utilised realistic hospital‐based case studies to demonstrate the impact of the framework and nonpharmacological resources. The health district funded the nonpharmacological resources, such as tactile activity interventions as described in Davis et al. (2023), assembled by local community groups. These resources support the application of the framework in routine practice.

### 3.2 Aim

To explore nurses' perceptions of the influence of a multifaceted nursing practice bundle, and the factors affecting practice change over time.

## 4 Methods

### 4.1 Design

A longitudinal qualitative approach was used to explore nurses' perceptions of the bundle's influence and the factors affecting nursing practice over time. Nurses were invited to participate in focus groups or interviews across two time points. The initial interviews and focus groups commenced six months post‐implementation of the bundle (May to June 2024), and the second round occurred nine months later (March to April 2025). Data collection time points were established in line with Cowie et al. (2020), who found that engagement with hospital‐based interventions may begin to deteriorate six months post‐implementation.

### 4.2 Theoretical Framework

This study was informed by Cohen‐Mansfield's unmet needs model (Cohen‐Mansfield 2000; Cohen‐Mansfield et al. 2015), which guided development of the bundle and supported the use of person‐centred, nonpharmacological interventions to address unmet needs in adults with cognitive impairment. The model also provided an interpretive lens for exploring nurses' experiences of translating theoretical concepts into practice within the hospital setting.

### 4.3 Study Setting and Recruitment

The bundle was implemented in November 2023 across four acute and subacute wards in rural and regional hospitals in Australia. Wards were purposively selected due to the high prevalence of patients with cognitive impairment. Enrolled and registered nurses (hereafter referred to collectively as nurses) were eligible to participate. Nurses who attended the bundle education were invited to participate via email. Nurses who completed the first data‐collection time point were subsequently invited to participate in the second.

Sampling was guided by the concept of information power, as described by Malterud et al. (2015). A sample of six to ten participants was considered adequate given the study's narrow aim, the specificity of the inclusion criteria, and the use of purposive maximum variation sampling (Palinkas et al. 2015). Intentionally recruiting nurses across different professional roles and sites ensured a diverse range of perspectives within a small sample, supporting in‐depth analysis and enhancing the transferability of findings. Information power was further strengthened through the application of an established theoretical framework (Cohen‐Mansfield 2000; Cohen‐Mansfield et al. 2015).

### 4.4 Data Collection

Data were collected using semi‐structured interviews and focus groups. Interview guides (File S2) were informed by factors influencing the implementation of healthcare innovations, as outlined by Windle et al. (2023): *i. People factors, ii. Innovation factors, iii. Organisational contexts, iv. Relational factors, v. Implementation process actions*. Preliminary analysis was undertaken following the first data collection time point, with findings informing refinement of questions for time point two, consistent with longitudinal qualitative approaches (Tuthill et al. 2020; Wanat et al. 2021). Nurses were offered the option to participate in either an individual interview or a focus group during their scheduled shift. This pragmatic approach was adopted to maximise participation across sites. All interviews and focus groups were conducted face‐to‐face by the first author (ERD), a Registered Nurse with experience in qualitative interviewing of healthcare clinicians.

#### 4.4.1 Researcher Positionality

Within this interpretive qualitative study, knowledge and meaning were understood as co‐constructed through the interaction between researcher and participant. The interviewer occupied an insider position as both a clinician‐researcher and colleague within the broader clinical and implementation context. This positioning provided contextual familiarity, a shared professional language, and an understanding of clinical workflows and organisational culture, which supported rapport and facilitated nuanced exploration of participants' experiences.

At the same time, the dual role may have influenced participant candour. Participants may have moderated criticism, emphasised favourable experiences, or assumed shared understandings due to collegial relationships, perceived professional expectations, or awareness of the interviewer's interest. The research team acknowledged that these relational dynamics formed part of the research context and may have shaped both the generation and interpretation of data. Rather than attempting to eliminate researcher influence, reflexivity was integral to the research process and informed both data collection and analysis.

### 4.5 Data Analysis

A longitudinal qualitative analysis, informed by the principles of reflexive thematic analysis (Braun and Clarke 2006), was conducted to address the study aim. Analysis occurred in three stages. Stages one and two incorporated cross‐sectional thematic analysis of the data collected at time point one and time point two. The third stage was a longitudinal comparative analysis, which examined similarities and differences across time points and informed the final themes presented in the findings (Figure 1). In this study, longitudinal analysis refers to comparisons across time points at the dataset level, rather than within individual change, consistent with longitudinal qualitative analysis approaches (Grossoehme and Lipstein 2016).

**FIGURE 1:**
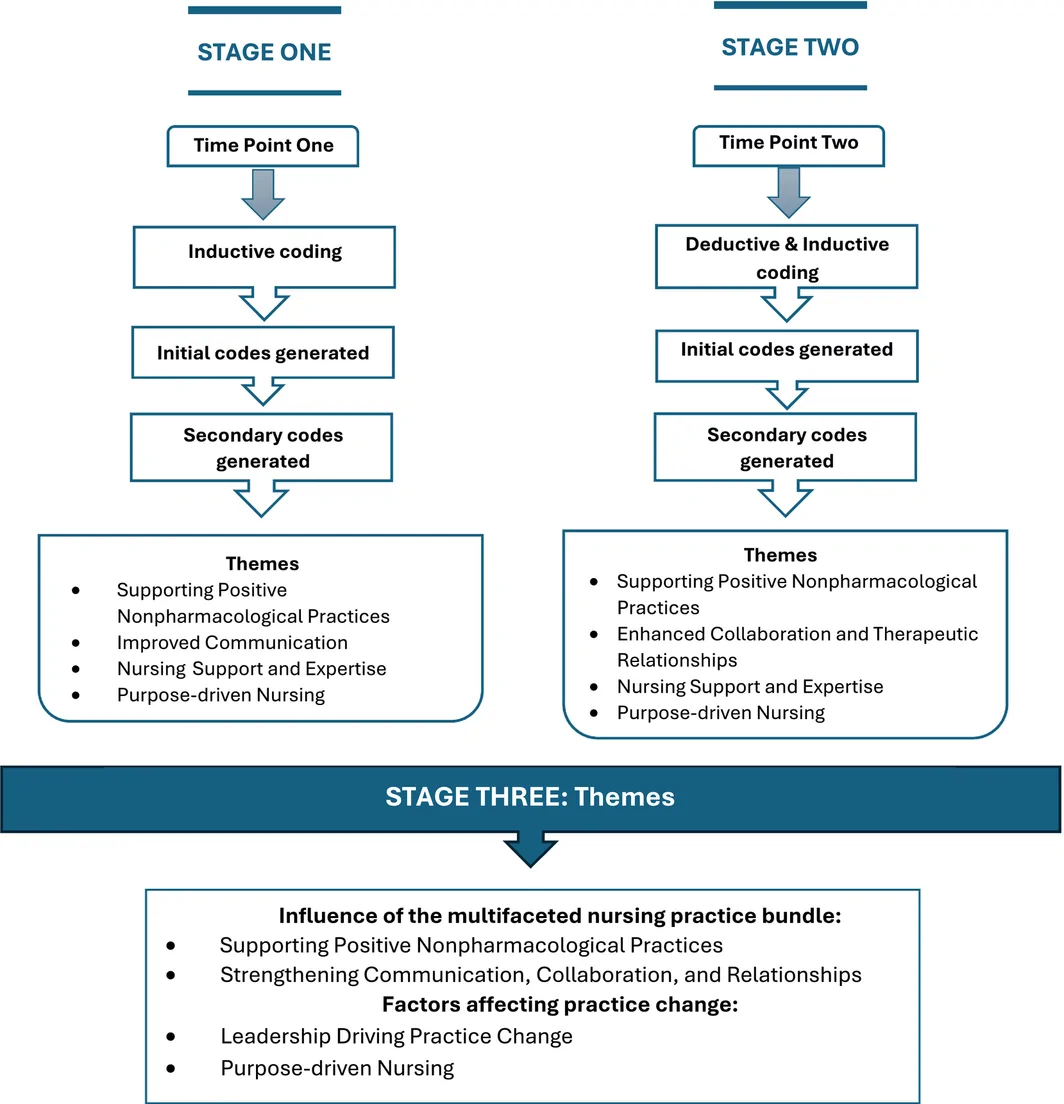
Three stages of data analysis. [Colour figure can be viewed at wileyonlinelibrary.com]

At time point one data were coded inductively. Initial coding was undertaken by the first author (ERD) followed by collaborative review and refinement by the full research team to develop preliminary themes. At time point two, a combined deductive‐inductive approach was used, in which existing codes informed analysis while allowing new codes to be generated as additional concepts emerged (Tuthill et al. 2020). Codes were organised into candidate themes, which were reviewed, refined, defined, and named through iterative team discussion. Stage one and stage two analyses are presented in File S3. The final stage of analysis involved a longitudinal synthesis in which the research team examined relationships between themes across time points to contextualise practice change and identify factors that affected it over time (Wanat et al. 2021).

Reflexivity was embedded throughout the analytic process to examine the researcher's influence on data interpretation. The researcher engaged in reflexive journaling and critical self‐reflection to examine how prior clinical experience, professional assumptions, and involvement in implementation may have shaped questioning, interpretation, and meaning‐making. Reflexive discussions within the broader research team and investigator triangulation were used to critically interrogate emerging interpretations, challenge assumptions arising from insider familiarity, and enhance analytic depth and credibility.

### 4.6 Ethical Considerations

Ethics approval was granted for this study (QA455; 2023/124). All participants received a Participant Information Statement and were informed that participation was voluntary, responses were anonymised, and that they could decline to answer any questions or withdraw from the study at any time without consequence. Written consent was obtained prior to participation in focus groups or interviews.

### 4.7 Rigour and Reflexivity

Rigour and reflexivity were addressed using the four principles of qualitative inquiry: credibility, transferability, dependability, and confirmability (Ahmed 2024). Credibility was maintained through investigator triangulation of field notes and interview transcripts, with an interdisciplinary research team comprising nurses (ERD, CA) and an occupational therapist (MB) bringing clinical, health management, and academic perspectives. All team members contributed to data coding, analysis, and interpretation, with deidentified transcripts provided to secondary coders (CA, MB) to reduce the risk of bias. Discrepancies were treated as opportunities for critical discussion and analytical curiosity, with the interdisciplinary research team collaboratively interrogating differing interpretations and underlying assumptions. Transferability was supported by purposive maximum variation sampling and by detailed contextual information about the study setting and participants. Dependability was strengthened by adherence to established methodological guidance for longitudinal qualitative research, informing study design and analytical processes (Tuthill et al. 2020; Wanat et al. 2021). Confirmability was reinforced through ongoing reflexive discussions during regular research team meetings. An audit trail was maintained to document analytic decisions and minimise bias. NVivo software was used to manage coding, with version histories retained to capture the iterative development and refinement of codes and themes. Transparency and reporting rigour were further supported by completing the Consolidated Criteria for Reporting Qualitative Research (COREQ) (File S4) (Tong et al. 2007).

## 5 Findings

### 5.1 Characteristics of Participants

Participants included Nurse Unit Managers (NUMs), Clinical Nurse Educators (CNEs), Registered Nurses (RNs), and Enrolled Nurses (ENs). At time point one, 17 nurses participated in four focus groups and one individual interview. At time point two, eight nurses participated in two focus groups and four individual interviews (Table 1). Attrition at time point two primarily reflected workforce turnover, with participants no longer employed within the health district or participating wards (*n* = 4), on extended leave (*n* = 2), or not responding to the follow‐up invitation (*n* = 3). Across both time points, interviews and focus groups had mean durations of 31 min (range: 20–47 min) and 33 min (range: 29–42 min).

**TABLE 1:** First and second round participant demographics across all sites.

	Time point one (*N* = 17)				Time point two (*N* = 8)			
	Focus group		Interview		Focus group		Interview	
Site 1	*N* = 5	CNE (*n* = 1)	*N* = 1	EN (*n* = 1)			*N* = 2	RN (*n* = 1) EN (*n* = 1)
		RN (*n* = 3)						
		EN (*n* = 1)						
Site 2	*N* = 4	CNE (*n* = 1)					*N* = 2	RN (*n* = 1) EN (*n* = 1)
		RN (*n* = 2)						
		EN (*n* = 1)						
Site 3	*N* = 4	NUM (*n* = 1)			*N* = 2	RN (*n* = 2)		
		CNE (*n* = 1)						
		RN (*n* = 2)						
Site 4	*N* = 3	RN (*n* = 1)			*N* = 2	RN (*n* = 1) EN (*n* = 1)		
		EN (*n* = 2)						

### 5.2 Perceived Influence on Nursing Practice and Factors Affecting Practice Change

Analysis of nurses' accounts of the bundle and factors affecting practice change over time revealed four themes. The first two themes, *Supporting positive nonpharmacological practices* and *Strengthening communication, collaboration, and relationships*, reflect perceived changes in nursing practice following bundle implementation. The remaining two themes, *Leadership driving practice change* and *Purpose‐driven nursing*, represent the contextual and organisational factors affecting practice change over time. Themes are presented with illustrative quotes from both data collection time points. Quotations are identified using participant numbers with an ‘a’ or ‘b’ suffix to denote time point one (e.g., P1a) or time point two (e.g., P1b).

#### 5.2.1 Influence on Nursing Practice

Nurses described ongoing positive changes to their nursing practice following implementation of the bundle. Two themes captured these changes: *supporting positive nonpharmacological practices* and *strengthening communication, collaboration, and relationships*.

##### 5.2.1.1 Supporting Positive Nonpharmacological Practices

Across both time points, nurses reported that the bundle supported more consistent use of nonpharmacological, person‐centred practices. Beyond facilitating the uptake of these practices, the findings suggest that the bundle may have contributed to a shift in clinical reasoning and understanding. Nurses described moving from reactive management of behaviours towards earlier recognition and proactive, person‐centred responses.

We need to be aware of…that subtle cry for help. (P14a)

If they are becoming agitated, I think oh, do they need to go to the toilet? What needs do they have? (P11b)

This really directed me to look at the person—our patient—their life story and their preferences for treatment. I've become a more person‐centred clinician. (P10b)

The framework appeared to strengthen clinical decision‐making by providing a structured approach to assessment and intervention, reducing reliance on ad hoc responses and supporting more consistent nonpharmacological care.

Without having the framework, they were just spur of the moment ideas, but having the framework is very helpful for an ideas base. (P11b)

Having the resources now is a lot better for us. Just having a set area where we can go and having the framework. Whereas before it was, you know, we're running off our own clinical judgement, and you know, have you exhausted every avenue? Yeah, we have. But how do you know? (P16b)

I think we were using them [nonpharmacological interventions], but inconsistently. There was no structure to what we were using before. It would be later that we'd try it. We'd struggle for days before we actually engaged (P1a).

Improved understanding of the underlying causes of behaviours that challenge influenced nurses' decision‐making regarding pharmacological interventions. Across both time points, nurses reported a reduced reliance on medication as a first‐line response, instead placing greater emphasis on identifying and addressing unmet needs relating to comfort, routine, and basic care.

I don't immediately think of [droperidol] or any of the medications. I think, okay, what's going on for this person, rather than how can I stop this behaviour? (P14a)

Before the implementation, I often heard nurses mention, ‘Oh, what medications can we give?’ That's the first line of management. Normally, nowadays, people care more about their sleep, their food, their clothing, and their comfort. It's really basic human needs they're considering more, rather than the medication management. So this is a big change. They're increasingly talking about their human needs. (P10b)

##### 5.2.1.2 Strengthening Communication, Collaboration, and Relationships

Nurses described progressive improvements in communication, collaboration, and relationships between the nursing team, patients, and carers following bundle implementation. Over time, the focus shifted from improved communication at time point one to deeper collaboration and relational engagement at time point two. Early changes in language and communication were associated with reduced stigma and bias towards patients with cognitive impairment, creating a foundation for more collaborative and relational care. These findings suggest that the bundle influenced communication and the broader culture of care for patients with cognitive impairment.

Participants highlighted the adoption of more professional, person‐centred terminology, which reframed how patients' behaviours were understood and discussed within teams. This shift supported more respectful communication and shaped nurses' perceptions of patients with cognitive impairment.

I think there's more use of the terminology of cognitively impaired as opposed to derogatory terms. Their behaviours aren't that they behave badly, but they don't have the cognition to support good behaviours…I've seen a change in that in the last 12 months. We talk about cognitive load and how we can decrease cognitive load or support them whilst they've got a heavy cognitive load. So, all that conversation, I think, has led to a more professional vocabulary, which I think changes the way we think about people. (P14b)

These changes appeared to influence clinical decision‐making by encouraging nurses to seek contextual information and to consider psychosocial or environmental factors contributing to distress.

#### 5.2.2 Factors Affecting Practice Change

Nurses' perceptions of factors influencing practice change over time revealed two themes: *leadership driving practice change* and *purpose‐driven nursing*. These themes were reinforced from time point one to time point two, with coding revealing consistent thematic patterns across both datasets.

##### 5.2.2.1 Leadership Driving Practice Change

Leadership from managers and champions was reported to drive practice change by reinforcing engagement with the bundle across both time points. This leadership appeared important in integrating the bundle into routine practice and shaping broader ward culture around care for patients with cognitive impairment. In addition, management supported implementation by ensuring the ongoing availability of resources, which were identified as a key enabler—or potential barrier—to ongoing use of the bundle in practice.

We've got really supportive NUMs [Nursing Unit Managers], but if we ever had someone who didn't see the value in it, they may not replenish all those resources. That could be challenging, but right now we've got that. So that's really easy to keep it going. (P1b)

Senior nurses described how they guided staff behaviours, expectations, and clinical norms through role modelling, reinforcement, and ongoing reference to the framework in daily care.

As a senior staff member, I try to refer to the framework, and then it reminds [nurses] there is a tool available. (P10b)

We can use [the framework] as a resource, like, hey, have you looked at this? This is how we actually operate in this ward. Having that expectation and standard that this is what we do here. (P1a)

Over time, use of the bundle appeared to become an expected standard of practice rather than an optional strategy, supporting more consistent person‐centred and nonpharmacological approaches across the team.

##### 5.2.2.2 Purpose‐Driven Nursing

Purpose‐driven nursing, underpinned by professional identity and personal values, was identified as a key factor influencing practice change over time. Despite challenges, including time pressures, staffing shortages, and acute care demands, nurses remained motivated to align their practice to the bundle because it reflected their commitment to provide meaningful, high‐quality care.

Nurses described how their use of the bundle was associated with improved patient experiences, thereby reinforcing ongoing engagement and shaping clinical decision‐making. Person‐centred, nonpharmacological approaches were perceived to reduce patient distress, facilitate task completion, and support therapeutic relationships, strengthening nurses' sense of professional fulfilment.

At the end of the day, we want to leave our shift feeling that we've implemented some positive activities throughout the course of the day and feeling good about what we've achieved. Being able to provide quality time for patients or improve their experience in that shift is important to all of us. (P12a)

I guess the motivation is that it makes their life better, the patient, and it makes my life easier because we are so busy. It's an all‐around win…my main incentive is to give good patient care. (P2b)

## 6 Discussion

This longitudinal qualitative study explored nurses' perceptions of a multifaceted nursing practice bundle designed to support hospitalised adults with cognitive impairment, and the factors affecting practice change over time. Findings suggest perceived improvements in nursing practices that align with Australian (Nursing and Midwifery Board of Australia 2016; ACSQHC 2021) and international nursing standards (International Council of Nurses 2021), particularly through increased use of *nonpharmacological practices* and *strengthened communication, collaboration, and relationships*. *Leadership driving practice change* and *purpose‐driven nursing* were identified as important factors affecting ongoing practice change.

The bundle appeared to drive changes in nursing practice, with care shifting from reactive to proactive, person‐centred approaches. This was reflected in earlier assessment of underlying causes, with nurses increasingly interpreting behaviours as expressions of unmet need, such as pain, distress, or environmental triggers. Improvements in language and communication among nurses further reinforced this shift, with the use of more professional, person‐centred terminology indicating a change in how adults with cognitive impairment are perceived (Goddu et al. 2018). This shift was facilitated by education informed by Cohen‐Mansfield's (2000) and Cohen‐Mansfield et al.'s (2015) unmet needs model. The findings extend the model beyond a theoretical explanation of behaviours that challenge by illustrating how understanding and recognition of unmet needs can influence clinical decision‐making for real‐world impact. Integration into practice was supported by an implementation framework and accessible nonpharmacological resources, allowing for structured yet adaptable approaches across patients and organisational contexts. This aligns with evidence supporting multifaceted interventions to overcome barriers in complex health settings (Jesto et al. 2024; Fontaine et al. 2024). Collectively, these changes may have important implications for patient outcomes, evident in nurses' accounts of improved patient experiences, including reduced distress, enhanced emotional safety, and more therapeutic engagement with care, consistent with evidence linking person‐centred approaches to improved outcomes (Ma et al. 2020; Carrarini et al. 2021; Kuske et al. 2021; James et al. 2023).

Examining factors influencing nursing practice change over time highlighted the importance of leadership and values alignment. Leadership and nursing champions were identified as supporting practice change by reinforcing expectations, facilitating access to resources, and modelling person‐centred care, consistent with implementation literature (Abbott et al. 2022; Davis et al. 2025; Windle et al. 2023). Purpose‐driven nursing, grounded in professional values of caring and dignity, further strengthened motivation to sustain practice change despite ongoing organisational pressures. Alignment between nursing values and practice has been associated with occupational satisfaction and improved care quality, whereas misalignment may contribute to moral distress (Midtbust et al. 2022; Watson 1988). Consistent with Shaban et al. (2024), improved patient experience and therapeutic relationships appeared to provide intrinsic validation, legitimising the bundle and supporting its continued use. These reinforcing mechanisms contributed to the integration of person‐centred nonpharmacological interventions within routine practice, suggesting a broader shift in care culture towards more needs‐based approaches. Together, these findings indicate that the multifaceted nature of the bundle—integrating a framework, education, and nonpharmacological resources—was important in supporting person‐centred nursing practice for adults with cognitive impairment in hospital settings.

### 6.1 Strengths and Limitations of the Work

A key strength of this study was its longitudinal qualitative design, which enabled exploration of the influence of the bundle and factors affecting practice change over time. Inclusion of nurses as participants enhanced the clinical relevance of the findings and strengthened the applicability of the results to real‐world nursing practice. Repeated data collection supported rapport building with participants and facilitated a deeper exploration of nurses' experiences.

Conversely, participants may have moderated their criticism due to collegial relationships with the interviewer and awareness of the interviewer's role. This was acknowledged at the outset of each session, with participants encouraged to speak openly and honestly, including sharing critical perspectives. Potential bias was further mitigated through reflexive practice, investigator triangulation, and collaborative analysis by an interdisciplinary research team.

Several other limitations should also be noted. Lower participation at the second time point reflects the transient nature of the healthcare workforce, a common challenge in longitudinal qualitative studies (Grossoehme and Lipstein 2016). To address this, purposive sampling and a recurrent cross‐sectional analytic approach were used, allowing each time point to be analysed as a standalone dataset while retaining breadth of perspectives across the study. This approach ensured that data from participants who did not complete follow‐up were not excluded from analysis. Despite this, attrition may have led to an overrepresentation of more motivated nurses and potentially introduced survivorship bias, limiting the generalisability of practice change among the broader population (Czeisler et al. 2021). Additionally, the authors acknowledge the absence of objective implementation fidelity data, including measures of exposure and consistency of ward‐level implementation.

### 6.2 Recommendations for Further Research

Future research should examine the long‐term outcomes of multifaceted nursing practice bundles across diverse healthcare settings to better understand the factors that support nursing practice and enhance transferability. Incorporating objective measures, such as antipsychotic medication use, behavioural emergency (code black) incidents, and falls, would strengthen the evidence base by quantifying the impact of these interventions on patient and system outcomes. Additionally, further qualitative research should incorporate the perspectives of patients and carers to determine whether observed changes in nursing practice translate into meaningful improvements in patient experience and care.

### 6.3 Implications for Policy and Practice

Findings from this study provide evidence to inform the design and implementation of organisational initiatives to improve nursing practices for hospitalised adults with cognitive impairment. The principles underpinning the bundle may be applicable across a range of clinical contexts where person‐centred, nonpharmacological approaches are required. Organisational investment in leadership, education, and accessible resources appears to support practice change and the integration of these approaches into routine care.

## 7 Conclusion

This is the first study to use a longitudinal qualitative methodology to explore nurses' perceptions of a multifaceted nursing practice bundle designed to support hospitalised adults with cognitive impairment. Findings suggest that combining an implementation framework, education, and nonpharmacological resources may support improvements in nursing practice. Over time, these changes appear to be affected by organisational support, leadership engagement, and education that reinforces nurses' understanding of unmet needs and the value of person‐centred, nonpharmacological interventions. These findings highlight the importance of aligning organisational structures with nursing values to support high‐quality, person‐centred care for adults with cognitive impairment in hospital settings.

## Author Contributions

**Christina Aggar:** conceptualisation (equal); methodology (equal); writing – review and editing (equal); formal analysis (supporting); writing – original draft (supporting). **Michelle Bissett:** conceptualisation (equal); methodology (equal); writing – review and editing (equal); formal analysis (supporting); conceptualisation (supporting); writing – original draft (supporting). **Erin Rachel Davis:** writing – original draft (lead); formal analysis (lead); conceptualisation (equal); methodology (equal); writing – review and editing (equal).

## Funding

The authors have nothing to report.

## Conflicts of Interest

The authors declare no conflicts of interest.

## Supporting information


**File S1:** The health district‐initiated implementation framework.

**File S2:** Interview guides from both time points.

**File S3:** Stage One and Stage Two data analysis codes and themes.

**File S4:** The Consolidated Criteria for Reporting Qualitative Research (COREQ).

## Data Availability

The data are not publicly available due to privacy or ethical restrictions.
