# Supplementary material for: Influence of a Multifaceted Nursing Practice Bundle to Support Hospitalised Adults With Cognitive Impairment: A Longitudinal Qualitative Study
Source: J Clin Nurs. 2026 Jun 22;35(11):5006–15. doi: 10.1111/jocn.70404 (PMC13629264; doi:10.1111/jocn.70404)
Supplement: Supplementary file 1 [file JOCN-35-5006-s001.docx]

Supplementary File 2: Interview guides from both time points

**Time Point One – Overview of questions**

Opening questions

- How long have you been working with people with cognitive impairments?
- How familiar were you with distraction activities before the framework rollout?

Introductory/ Transition questions

- Since the introduction of the Distraction Activity Framework, can you think of a time when you attempted to implement an activity to care for a patient with cognitive impairment? How well did this go?

Key questions regarding implementation and effect

*These questions are around any perceived benefits or barriers for the patient.*

- What, if any, were the barriers or challenges to implementing the distraction activities?
- Were there any safety concerns observed during implementation of distraction activities?
- What, if any, were the benefits of implementing distraction activities?

*Im going to ask another set of questions now, this is more around your view of the activities and how useful the framework was as a tool.*

Key Questions regarding personal beliefs

- Since the introduction of the Distraction Activity Framework and resources, how has your perception on caring for people with cognitive impairment changed? If at all.
- Did you find the distraction activity framework useful or not useful when implementing distraction activities?
- If given the opportunity, would you continue using the distraction activity framework as a tool to care for patients with cognitive impairment? Why or why not?

Ending questions

- What elements of the program could be enhanced?
- Is there anything else that would assist you in using the distraction activity framework?

**Time point two – Overview of questions**

1. How, if at all, has the distraction activity program changed your nursing care practice for adults with cognitive impairment?

| **If So**   - How have these changes in your practice impacted patient care or patient outcomes? - What motivates you to continue using the distraction activity framework resources? | **If Not**   - Can you elaborate on this - Is there anything that would motivate you to use the distraction activity framework resources? |
| --- | --- |

1.

2. What is your sense of the impact of the program on patients and/or family members? Please share some of their feedback….

3. Since the introduction of the program, have you looked for or participated in any additional training to improve your skills in supporting adults with cognitive impairment?

| **If So**   - What kind of training or education was this, and did this training further influence your approach to supporting adults with cognitive impairment? | **If Not**   - Is there any reason for this? Please elaborate. |
| --- | --- |

4.

5. In your experience overall, has the Distraction Activity Framework program continued to be used on your ward?

| **If So**   - What elements of the program are used and how? - What factors have supported ongoing use? | **If Not**   - What challenges or barriers have prevented its ongoing use? |
| --- | --- |

6. Can you describe the availability of distraction activity resources on your ward?

7. What do you believe is needed to sustain the program, in terms of supports and resources?

8. Can you tell me about the broader health priorities on the ward over the past 12 months.

9. How does the program align (or not) with the other priorities in NSW health?
