# Supplementary material for: Influence of a Multifaceted Nursing Practice Bundle to Support Hospitalised Adults With Cognitive Impairment: A Longitudinal Qualitative Study
Source: J Clin Nurs. 2026 Jun 22;35(11):5006–15. doi: 10.1111/jocn.70404 (PMC13629264; doi:10.1111/jocn.70404)
Supplement: Supplementary file 2 [file JOCN-35-5006-s002.docx]

Supplementary File 1. The health district-initiated implementation framework

*Resource developed by Allison Wallis and Karen Bowen for the Northern New South Wales Local Health District. Used with permission.*

Northern New South Wales Local Health District. (2022). *Distraction Activity Framework for Patients with a Cognitive Impairment.* [Unpublished internal document]. Author
