# Supplementary material for: Influence of a Multifaceted Nursing Practice Bundle to Support Hospitalised Adults With Cognitive Impairment: A Longitudinal Qualitative Study
Source: J Clin Nurs. 2026 Jun 22;35(11):5006–15. doi: 10.1111/jocn.70404 (PMC13629264; doi:10.1111/jocn.70404)
Supplement: Supplementary file 3 [file JOCN-35-5006-s003.docx]

Supplementary File 4: The Consolidated Criteria for Reporting Qualitative Research (COREQ)

| **No** | **Item** | **Guide questions/description** | **Answer** |
| --- | --- | --- | --- |
| **Domain 1: Research team and reflexivity** | | | |
| Personal Characteristics | | | |
| 1. | Interviewer/facilitator | Which author/s conducted the interview or focus group? | ERD |
| 2. | Credentials | What were the researcher's credentials? *E.g. PhD, MD* | RN, PhD candidate |
| 3. | Occupation | What was their occupation at the time of the study? | Registered Nurse, Nursing Lecturer |
| 4. | Gender | Was the researcher male or female? | Female |
| 5. | Experience and training | What experience or training did the researcher have? | Previous experience conducting qualitative interviews as a research assistant.  2-day online focus group workshop (ACSPRI), previous experience conducting interviews for qualitative research |
| Relationship with participants | | | |
| 6. | Relationship established | Was a relationship established prior to study commencement? | Worked within the health district but was not currently working as a Registered Nurse on any of the participating wards. |
| 7. | Participant knowledge of the interviewer | What did the participants know about the researcher? e*.g. personal goals, reasons for doing the research* | Participants understood that this research was conducted as part of the interviewer's thesis but were unaware of the overall thesis aim. |
| 8. | Interviewer characteristics | What characteristics were reported about the interviewer/facilitator? e.g. *Bias, assumptions, reasons and interests in the research topic* | Working as a clinician in the district supported rapport and contextual understanding but also required ongoing reflexivity to minimise potential bias and ensure participants felt comfortable sharing their perspectives openly. |
| **Domain 2: study design** | | | |
| Theoretical framework | | | |
| 9. | Methodological orientation and Theory | What methodological orientation was stated to underpin the study? *e.g. grounded theory, discourse analysis, ethnography, phenomenology, content analysis* | Longitudinal qualitative approach (Tuthill et al., 2020; Wanat et al., 2021), supported by the Theory of Unmet Needs (Cohen-Mansfield et al., 2015). |
| Participant selection | | | |
| 10. | Sampling | How were participants selected? *e.g. purposive, convenience, consecutive, snowball* | Purposeful sampling |
| 11. | Method of approach | How were participants approached? e*.g. face-to-face, telephone, mail, email* | Email |
| 12. | Sample size | How many participants were in the study? | 17 participants |
| 13. | Non-participation | How many people refused to participate or dropped out? Reasons? | Nine participants did not participate in the second-round data collection. Six of these participants no longer worked on an included ward or were on extended periods of leave. The remaining three participants did not respond, with no reason given. |
| Setting | | | |
| 14. | Setting of data collection | Where was the data collected? e*.g. home, clinic, workplace* | Within the workplace. |
| 15. | Presence of non-participants | Was anyone else present besides the participants and researchers? | No |
| 16. | Description of sample | What are the important characteristics of the sample? *e.g. demographic data, date* | Demographic data: occupation and site.  Focus group or interview participation. |
| Data collection | | | |
| 17. | Interview guide | Were questions, prompts, guides provided by the authors? Was it pilot tested? | Yes. An initial interview was conducted to pilot test the questions. The results of this were included in the study. |
| 18. | Repeat interviews | Were repeat interviews carried out? If yes, how many? | Yes. There was one interview and four focus groups conducted at time point one. At time point two, there were two focus groups and five interviews. |
| 19. | Audio/visual recording | Did the research use audio or visual recording to collect the data? | Audio recording |
| 20. | Field notes | Were field notes made during and/or after the interview or focus group? | Yes, during and after. |
| 21. | Duration | What was the duration of the interviews or focus group? | Time point one: Averaging 31 minutes (20 minutes – 47 minutes)  Time point two: 33 minutes (29 minutes – 42 minutes) |
| 22. | Data saturation | Was data saturation discussed? | No. Information power is a preference in this study. |
| 23. | Transcripts returned | Were transcripts returned to participants for comment and/or correction? | No |
| **Domain 3: analysis and findings** | | | |
| Data analysis | | | |
| 24. | Number of data coders | How many data coders coded the data? | Three |
| 25. | Description of the coding tree | Did authors provide a description of the coding tree? | Yes |
| 26. | Derivation of themes | Were themes identified in advance or derived from the data? | Both |
| 27. | Software | What software, if applicable, was used to manage the data? | NVivo 14 |
| 28. | Participant checking | Did participants provide feedback on the findings? | No |
| Reporting | | | |
| 29. | Quotations presented | Were participant quotations presented to illustrate the themes/findings? Was each quotation identified? e*.g. participant number* | Yes |
| 30. | Data and findings consistent | Was there consistency between the data presented and the findings? | Yes |
| 31. | Clarity of major themes | Were major themes clearly presented in the findings? | Yes |
| 32. | Clarity of minor themes | Is there a description of diverse cases or discussion of minor themes? | No |
