# Supplementary material for: Influence of a Multifaceted Nursing Practice Bundle to Support Hospitalised Adults With Cognitive Impairment: A Longitudinal Qualitative Study
Source: J Clin Nurs. 2026 Jun 22;35(11):5006–15. doi: 10.1111/jocn.70404 (PMC13629264; doi:10.1111/jocn.70404)
Supplement: Supplementary file 4 [file JOCN-35-5006-s004.docx]

Supplementary File 3: Stage One and Stage Two data analysis codes and themes

| **Influence of the multifaceted nursing practice bundle** | | | | | | | |  |  |
| --- | --- | --- | --- | --- | --- | --- | --- | --- | --- |
| **Phrases in bold represent common codes and themes across both time points* | | | | | | | |  |  |
| **FIRST TIME POINT** | | | | | **SECOND TIME POINT** | | |  |  |
| **Initial Coding** | | **Secondary Coding** | **Themes** | | **Initial Coding** | **Secondary Coding** | **Themes** |  |  |
| **Discussions between staff** | | Interprofessional communication | Improved Communication | | Clear goals in caring for patients | Collaborative Teamwork | Enhanced Collaborative and Therapeutic Relationships |  |  |
| **Documentation** | |  |  |  | **Discussions between staff** |  |  |  |  |
| Engagement with external stakeholders | |  |  |  | **Documentation** |  |  |  |  |
| **Enhanced collaboration with carers** | |  |  |  | **Enhanced collaboration with carers** |  |  |  |  |
| Team leaders directing staff | |  |  |  | Multidisciplinary approach |  |  |  |  |
| **Staff handover** | |  |  |  | **Staff handover** |  |  |  |  |
| Communication between patients and staff | | Patient communication |  |  | Team nursing |  |  |  |  |
| **Communication with patients** | |  |  |  | Increase in professional and person-centred vocabulary | Professional Communication |  |  |  |
| Potential to improve carers and patient communication | |  |  |  | **Communication with patients** | Building Therapeutic Relationships |  |  |  |
|  |  |  |  |  | Facilitates relationship between patients and family |  |  |  |  |
| **Accessibility of resources** | | **Engagement with Nonpharmacological Interventions** | **Supporting Positive Nonpharmacological** | | Rapport building with patients |  |  |  |  |
| **Initiating nonpharmacological interventions early** | |  |  |  | Improved patient and clinician relationships |  |  |  |  |
| Lends credibility to nonpharmacological suggestions | |  |  |  | **Accessibility of resources** | **Engagement with Nonpharmacological Interventions** | **Supporting Positive Nonpharmacological** |  |  |
| More consistent use of nonpharmacological interventions | |  |  |  | **Initiating nonpharmacological interventions early** |  |  |  |  |
| **Now routine use of nonpharmacological interventions** | |  |  |  | Not able to hang framework on the wall |  |  |  |  |
| **Provides ideas for nonpharmacological interventions** | |  |  |  | Framework not readable from a distance |  |  |  |  |
| Structures nonpharmacological interventions | |  |  |  | **Now routine use of nonpharmacological interventions** |  |  |  |  |
| Supports nonpharmacological intervention implementation | |  |  |  | Positive experiences with nonpharmacological interventions impact future use |  |  |  |  |
| Avoiding the use of sedatives | | Critical Thinking and Reflection |  |  | Previous negative experiences shape future interventions |  |  |  |  |
| Building on preexisting knowledge and skills | |  |  |  | Nurses are more aware of the benefits of nonpharmacological interventions |  |  |  |  |
| Identify behaviour and management strategy | |  |  |  | **Provides ideas for nonpharmacological interventions** |  |  |  |  |
| Nonpharmacological interventions to reduce the use of pharmacological interventions | |  |  |  | The framework isn't routinely used |  |  |  |  |
| Nurses are more aware of positive care practices | |  |  |  | Trying multiple nonpharmacological interventions |  |  |  |  |
| Nurses thinking enhanced in areas they were struggling | |  |  |  | Business as usual | Critical Thinking and Care Practice |  |  |  |
| The framework is used in conjunction with nursing assessment | |  |  |  | Delaying pharmacological intervention |  |  |  |  |
| **A variety of available nonpharmacological interventions** | | **Personalising Interventions** |  |  | Earlier intervention to prevent escalation |  |  |  |  |
| **Considering the patients’ needs** | |  |  |  | Higher use of preexisting person-centred care tools |  |  |  |  |
| Families are beneficial when present | |  |  |  | Impact on nursing care practice |  |  |  |  |
| Family can be a calming influence | |  |  |  | Considering the longer-term impact of medication |  |  |  |  |
| Family collaboration to individualise care plans is needed | |  |  |  | Nurses exploring deeper patient needs when nonpharmacological interventions don't work |  |  |  |  |
| **Family involvement** | |  |  |  | **A variety of available nonpharmacological interventions** | **Personalising Interventions** |  |  |  |
| Personalised care in consultation with the family | |  |  |  | **Considering the patients’ needs** |  |  |  |  |
| Knowing the person and tailoring care | |  |  |  | **Family involvement** |  |  |  |  |
| More personalised interventions | |  |  |  | Family not being included |  |  |  |  |
| **Person-centred care** | |  |  |  | Familiarity with the patient |  |  |  |  |
| Patient behaviour may prompt particular nonpharmacological interventions | |  |  |  | One-on-one conversations |  |  |  |  |
|  | |  |  | | Patient behaviour may prompt particular nonpharmacological interventions |  |  |  |  |
|  |  |  |  |  | Patient interest |  |  |  |  |
|  |  |  |  |  | **Person-centred care** |  |  |  |  |
|  |  |  |  |  | Repeating proven nonpharmacological interventions |  |  |  |  |
|  |  |  |  |  | Working with the patient |  |  |  |  |
|  |  |  |  |  | Adhering to the national standards | Complying with Organisational Expectations |  |  |  |
|  |  |  |  |  | Aligns with organisational key performance indicators |  |  |  |  |
|  |  |  |  |  | An organisational emphasis on positive care practices |  |  |  |  |
|  |  |  |  |  | Avoiding the use of sedatives/ medication |  |  |  |  |
|  |  |  |  |  | Infection control concerns |  |  |  |  |
|  |  |  |  |  | Works in combination with preexisting guidelines |  |  |  |  |
| **Factors affecting practice change** | | | | | | | |  |  |
| **Phrases in bold represent common codes and themes across both time points* | | | | | | | |  |  |
| **FIRST TIME POINT** | | | | | **SECOND TIME POINT** | | |  | **Initial Coding** |
| **Initial Coding** | **Secondary Coding** | | | **Themes** | Adhoc education between staff | **Secondary Coding** | **Themes** |  |  |
| **Dedicated staff** | **Leadership and Champions** | | | **Nursing Support and Expertise** | Being directed by medical staff | **Leadership and champions** | **Nursing Support and Expertise** |  |  |
| **Staffing changes** |  |  |  |  | Dedicated staff |  |  |  |  |
| Experience |  |  |  |  | Nurse inclusion in selecting available resources |  |  |  |  |
| **Positive experiences with nonpharmacological interventions impact future use** |  |  |  |  | Organisational support |  |  |  |  |
| Staff champion |  |  |  |  | Prompts from specialist staff |  |  |  |  |
| Staff member bringing in resources |  |  |  |  | Providing education to senior staff |  |  |  |  |
| Staff knowledge and skills | **Nurses Understanding** | | |  | Staff champion |  |  |  |  |
| Understanding the effect of cognitive impairment on the person |  |  |  |  | Supportive managers |  |  |  |  |
| Using knowledge to mitigate risks |  |  |  |  | Additional programs running to support nonpharmacological interventions |  |  |  |  |
| Education needs to capture staff members across multiple shifts | **Support for Staff** | | |  | Nurse are familiar with pathways to guide care | **Nurses Understanding** |  |  |  |
| Guides nurses who are struggling to help the patient |  |  |  |  | Familiarity with the program |  |  |  |  |
| Nurses feel historically unsupported |  |  |  |  | Nurses rely on their own knowledge |  |  |  |  |
| **Support for new staff members** |  |  |  |  | Perception that current practice doesn't benefit from the framework |  |  |  |  |
| Supports nurses when they are busy |  |  |  |  | Accessibility of resources |  |  |  |  |
| Repeated education to capture new staff members |  |  |  |  | A variety of available nonpharmacological interventions | **Support for staff** |  |  |  |
| Volunteers |  |  |  |  | Dedicated space |  |  |  |  |
| Workplace Culture |  |  |  |  | Education through alternate occupation |  |  |  |  |
| **Conflicting acute care priorities** | **Impact on Nurses/ Shift** | | | **Purpose-driven Nursing** | High education saturation |  |  |  |  |
| Eases nursing workload |  |  |  |  | In depth education |  |  |  |  |
| Inadequate staffing |  |  |  |  | Physical layout of the ward |  |  |  |  |
| Nonpharmacological interventions support nurses to care for multiple patient |  |  |  |  | Mandatory education |  |  |  |  |
| Nurses’ perceptions of the impact on family |  |  |  |  | Meaningful education |  |  |  |  |
| Stress reduction for staff |  |  |  |  | Ongoing education |  |  |  |  |
| **Safety concerns** |  |  |  |  | Resource visibility |  |  |  |  |
| **Workload** |  |  |  |  | Resource visibility |  |  |  |  |
| Benefit to the patient’s mood | **Improved Patient Outcomes** | | |  | Self-directed education |  |  |  |  |
| **Keeps patients occupied** |  |  |  |  | Services and resources |  |  |  |  |
| Improving patients mental health |  |  |  |  | Staffing changes |  |  |  |  |
| **Positive impact on patients** |  |  |  |  | Support for new staff members |  |  |  |  |
| **Positive patient outcomes** |  |  |  |  | Volunteers |  |  |  |  |
| Providing patients a sense of purpose |  |  |  |  | Workplace education |  |  |  |  |
| **Reduction in challenging behaviour** |  |  |  |  | Additional assessment is perceived as a burden to nurses |  |  |  |  |
| Improved community perceptions of the care provided | **Nurses' Sense of Purpose** | | |  | Aligns with ward needs | **Impact on Nurses/ Shift** | **Purpose-driven Nursing** |  |  |
| Improved family perceptions of nursing staff |  |  |  |  | **Conflicting acute care priorities** |  |  |  |  |
| Nursing goal to improve patients experience |  |  |  |  | Conflicting education priorities |  |  |  |  |
| Nursing goal to reduce hospital-acquired complications |  |  |  |  | Conflicting ward priorities |  |  |  |  |
| Personally rewarding |  |  |  |  | Eases nurse’s workload |  |  |  |  |
| Treating the patient as a loved one |  |  |  |  | Eases nurse’s workload |  |  |  |  |
|  | | | | | Enhances ability to perform tasks |  |  |  |  |
|  |  |  |  |  | Facilitates acute care investigations |  |  |  |  |
|  |  |  |  |  | Facilitates medical intervention |  |  |  |  |
|  |  |  |  |  | Improving staff retention |  |  |  |  |
|  |  |  |  |  | Lack of time |  |  |  |  |
|  |  |  |  |  | Periods of high stress |  |  |  |  |
|  |  |  |  |  | **Safety concerns** |  |  |  |  |
|  |  |  |  |  | **Workload** |  |  |  |  |
|  |  |  |  |  | **Keeps patients occupied** | **Improved Patient Outcomes** |  |  |  |
|  |  |  |  |  | **Positive impact on patients** |  |  |  |  |
|  |  |  |  |  | **Positive patient outcomes** |  |  |  |  |
|  |  |  |  |  | Reduction in challenging behaviour |  |  |  |  |
|  |  |  |  |  | Aligns with personal career goals | **Nurses' Sense of Purpose** |  |  |  |
|  |  |  |  |  |  |  |  |  |  |
